# Self-Assembly Control of Y-Series Non-fullerene Acceptors for Sustainable and Scalable Organic Photovoltaics

**DOI:** 10.1007/s40820-025-02021-7

**Published:** 2026-01-05

**Authors:** Dingqin Hu, Hua Tang, Jiehao Fu, Yaohui Li, Lei Liu, Peihao Huang, Jie Lv, Daming Zheng, Yakun He, Heng Liu, Baomin Xu, Zheng Hu, Xinhui Lu, Zeyun Xiao, Gang Li, Yang Michael Yang, Frédéric Laquai, Christoph J. Brabec, Duu-Jong Lee, Hsien-Yi Hsu

**Affiliations:** 1 Department of Materials Science and Engineering, School of Energy and Environment, Centre for Functional Photonics (CFP), City University of Hong Kong, Kowloon Tong, Hong Kong, People’s Republic of China; 2 Department of Mechanical Engineering, City University of Hong Kong, Kowloon Tong, Hong Kong, People’s Republic of China; 3 Institute of Materials for Electronics and Energy Technology (I-MEET), Friedrich-Alexander-Universität Erlangen-Nürnberg, Martensstrasse 7, 91058 Erlangen, Germany; 4 Helmholtz-Institute Erlangen-Nürnberg (HI ERN), Immerwahrstraße 2, 91058 Erlangen, Germany; 5 KAUST Solar Center, Physical Sciences and Engineering Division (PSE), King Abdullah University of Science and Technology (KAUST), 23955-6900 Thuwal, Kingdom of Saudi Arabia; 6 Department of Electronic and Information Engineering Research Institute for Smart Energy (RISE), The Hong Kong Polytechnic University Hung Hum, Kowloon, Hong Kong, 999077 People’s Republic of China; 7 State Key Laboratory of Modern Optical, Instrumentation College of Optical Science and Engineering, Zhejiang University, Hangzhou, 310027 Zhejiang People’s Republic of China; 8 Chongqing Institute of Green and Intelligent Technology, Chinese Academy of Sciences, Chongqing, 400714 People’s Republic of China; 9 Department of Physics, The Chinese University of Hong Kong, New Territories, Hong Kong, 999077 People’s Republic of China; 10 Department of Materials Science and Engineering, Southern University of Science and Technology, Shenzhen, 518055 People’s Republic of China; 11 Key Laboratory of Mesoscopic Chemistry of MOE and Jiangsu Provincial Laboratory for Nanotechnology, School of Chemistry and Chemical Engineering, Nanjing University, Nanjing, 210023 People’s Republic of China

**Keywords:** Organic solar cells, Self-assembly control, Large-area modules

## Abstract

Sustainability and scalability remain critical hurdles for the commercialization of organic solar cells (OSCs). However, addressing both poses challenge. Herein, we introduce a simple yet effective strategy utilizing 3,5-dichloropyridine (PDCC) as a solid additive to fine-tune the self-assembly behavior of Y-series non-fullerene acceptors (NFAs) to tackle the upscaling limitations in green-solvent-processed OSCs. PDCC predominantly interacts with Y-series NFAs, facilitating molecular crystallization and thereby driving the self-assembly of Y-series NFAs during film-forming dynamics, leading to more uniform active layers with improved molecular packing and reduced charge recombination. As a result, PDCC-driven self-assembly strategy enables high-performance OSCs with a power conversion efficiency (PCE) of 20.47%. When translated to sustainable fabrication, this strategy significantly boosts the PCE of large-area green-solvent-processed OSC modules (19.3 cm^2^) from 13.87% to 15.79%, ranking it among the best-performing green-solvent-processed large-area OSC modules (> 18 cm^2^). Beyond its impact on PCE enhancement, PDCC serves as a multifunctional additive to improve long-term stability and exhibits strong universality across multiple material systems. This work establishes a promising approach for advancing sustainable and scalable OSCs, paving the way for their commercialization.

**Supplementary Information:**

The online version contains supplementary material available at 10.1007/s40820-025-02021-7.

## Highlights

- The self-assembly behavior of Y-series non-fullerene acceptors and film formation dynamics are elucidated via in situ characterization, providing critical insights for sustainable and scalable organic solar cells (OSCs).
- A 3,5-dichloropyridine-assisted self-assembly strategy enables 20.47% efficiency for small-area OSCs and 15.79% for sustainable organic photovoltaic modules (19.3 cm^2^).
- This versatile self-assembly control approach is broadly applicable to various material systems, paving the way toward the commercialization of OSC.

**Supplementary Information:**

The online version contains supplementary material available at 10.1007/s40820-025-02021-7.

## Graphical abstract

**Figure:**
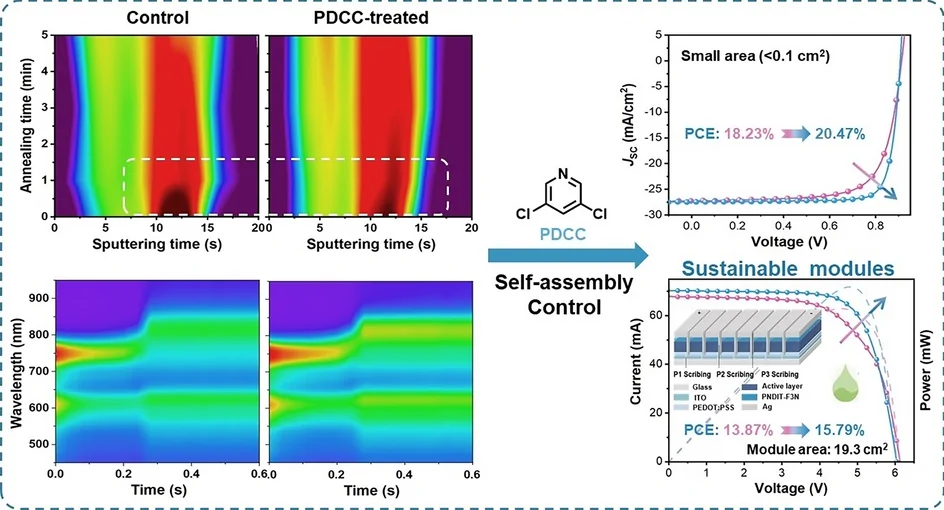

## Introduction

Organic solar cells (OSCs) have emerged as a promising next-generation photovoltaic technology, distinguished by their mechanical flexibility, tunable spectral absorption, and low environmental impact [1–14]. Recent advances in materials design and device engineering have propelled OSCs to power conversion efficiencies (PCEs) exceeding 20%, marking a critical transition toward commercialization [5–22]. However, researches about sustainable industrial production of highly efficient large-area OSCs are still lacking, primarily attributed to the significant challenge of maintaining uniform film-forming kinetics across large areas. Therefore, it is urgent to develop innovative strategies to regulate film formation processes, facilitating high-performance, stable, sustainable, and large-area productions fabricated toward commercialization.

Controlling the self-assembly of organic photovoltaic (OPV) materials has proven to be an effective strategy for tailoring the film formation process toward highly efficient and stable OSCs. Current approaches predominantly focus on molecular engineering, post-treatment techniques, ternary, and layer-by-layer (LBL) strategies [23–25]. For instance, Sun et al. developed three low-cost PTQ-derivative donor polymers through synergistic ternary copolymerization and side-chain engineering involving various benzothiadiazole (BT) units, enabling precise modulation of molecular self-assembly behavior. Among them, PTQ18, incorporating monofluorinated and monoalkoxy-substituted BT moieties, demonstrated optimal regulation of self-assembled morphology, leading to superior compatibility with Y-series non-fullerene acceptors (NFAs). As a result, PTQ18-based devices achieved a PCE of 19.68%, outperforming those based on PTQ17 (17.04%) and PTQ19 (18.50%) [26]. Likewise, Bo et al. has shown that improving the intermolecular connectivity of NFAs through molecular engineering is an effective strategy to realize hierarchically supramolecular self-assembly of NFAs [27]. This highlights the critical role of rational molecular design in governing the self-assembly and ultimately the performance of OSCs. Besides, device processing optimization provides an alternative pathway for regulating self-assembly morphology. For instance, Wang et al. developed hybrid post-processing strategy (thermal and solvent annealing) to achieving high-performance all-small-molecule (ASM) OSCs via controlling self-assembly active-layer morphology. Compared to w/o and thermal treatment, hybrid post-processing can effectively achieve face-on molecular orientation, resulting in more efficient photon harvest and charge transport [28]. This approach led to an outstanding PCE of 8.99% with enhanced fill factor (FF) from 68.62% to 72.21% [28]. Recently, Song et al. introduced a trimer-induced pre-swelling (TIP) strategy by synthesizing a twisted, three-dimensional star-shaped trimer (BTT-Out) and integrating it with a LBL deposition technique. In this approach, BTT-Out is incorporated into the buried D18 donor layer, enabling the fabrication of thick-film OSCs. Owing to its unique molecular configuration and spontaneous self-organization behavior, the BTT-Out trimer effectively pre-swells the D18 network, thereby promoting acceptor infiltration and accelerating donor–acceptor (D/A) interface formation. As a result, TIP-modified devices achieved a high PCE of 20.3% in thin films and 18.8% in thick films, alongside enhanced device stability, demonstrating the potential of this strategy to advance the commercial scalability of OSCs [29]. Despite notable advancements in tuning the self-assembly of active layers to improve device performance, most existing strategies remain intricate and lack compatibility with sustainable, large-scale manufacturing processes. It is worth mentioning that Y-series NFAs have emerged as pivotal materials in advancing OSCs toward commercialization [30]. In our previous work, we demonstrated that Y-series NFAs possess strong potential for regulating film-formation dynamics, thereby enabling high-efficiency and stable devices [31]. These findings underscore the urgent need to develop innovative self-assembly modulation strategies tailored specifically to Y-series NFAs—particularly those that are scalable and compatible with sustainable processing.

Herein, we report a simple yet effective strategy employing 3,5-dichloropyridine (PDCC) as a solid additive to regulate the self-assembly of Y-series NFAs molecules toward highly efficient, and stable OSCs. The incorporation of nitrogen atoms enables PDCC predominantly interacts with acceptor molecules, assisting *J*-aggregation and molecular crystallization. Under the drive of crystallization, improving the self-assembly of Y-series NFAs during film formation processes, result in well-define morphology and ordered molecular packing, promoting efficient exciton dissociation, charge transport, and suppressed recombination losses. As a result, PDCC-driven self-assembly strategy enables high-performance OSCs with a power conversion efficiency (PCE) of 20.47%. When translated to sustainable fabrication, this strategy significantly boosts the PCE of large-area green-solvent-processed OSC modules (19.3 cm^2^) from 13.87% to 15.79%, ranking it among the best-performing green-solvent-processed large-area OSC modules (> 18 cm^2^). Beyond its effectiveness in the PM6:BTP-eC9 system, PDCC-induced morphology control exhibits strong universality across other material systems, highlighting its broad applicability. Thus, this work establishes a promising approach for advancing industrial production of highly efficient OSCs and sustainable, large-area modules, paving the way for their commercialization.

## Experimental Section

### Materials

All reagents and solvents, unless otherwise specified, were purchased from Energy Chemical, Tansoole, Suna Tech, Aldrich, and JiangSu GE-Chem Biotech., Ltd. and were used without further purification. All materials are provided by commercial suppliers: PM6, Y6, BTP-eC9, L8-BO, PNDIT-F3N was purchased from Solarmer Energy Inc. The PDCC was purchased from Macklin. PEDOT:PSS (Clevios P VP AI. 4083) was purchased from Xi’an Yuri Solar Co., Ltd.

### Device Fabrication and Characterizations

#### Small-Area Device Fabrication

The device structures were ITO/PEDOT:PSS/Active layer/PNDIT-F3N/Ag. ITO coated glass substrates were cleaned with detergent water, deionized water, acetone, and isopropyl alcohol in an ultrasonic bath sequentially for 15 min, and further treated with UV exposure for 15 min in a UV-ozone chamber. A thin layer (ca. 30 nm) of PEDOT:PSS (Bayer Baytron 4083) was first spin-coated on the substrates with 4000 rpm and baked at 120 °C for 10 min under ambient conditions. The substrates were then transferred into a nitrogen-filled glove box. The PM6:Y6, PM6:BTP-eC9, PM6:L8-BO concentration was 16 mg mL^−1^ with D:A ratio of 1:1.2 (w/w) and PDCC 8 mg mL^−1^ in chloroform (CF) or o-xylene (*o*-XY) solution. The PM6:BTP-eC9 solution needs heat stirring with 40 °C/2 h, and the substrate heat treatment 80 °C/5 min (when *o*-XY as solvent). After spin-coating at 3000 rpm for 30 s, the blend films were thermal-annealed at 90 °C for 5 min. Then, PNDIT-F3N as the electron transporting layer was spin-coated on the active layer by 4000 rpm/30 s. Finally, the substrates were transferred to a thermal evaporator, and top electrode was evaporated at a pressure of 2 × 10^–5^ Pa.

#### Large-Area Device Fabrication

The pre-deposited ITO substrate was scribed by a 1064 nm nano-sec laser beam (2 W) to form an isolated ITO unites. After cleaning, PEDOT:PSS layer, PM6:BTP-eC9 without or with PDCC layer and PNDIT-F3N layer were sequentially deposited onto ITO substrate in the same way as the small area device. Next, the stacked layer was scribed by another 532 nm nano-sec laser beam (P2 scribing). Ag electrode was thermally deposited under a pressure of 3.3 × 10^−4^ Pa. P3 scribing (532 nm nano-sec laser beam) was carried out to form a series of sub-cells. The geometric fill factor (GFF) of the module is 97.0%. The module area used to measure the PCE was defined by the aperture mask as 19.3 cm^2^.

The external quantum efficiency (*EQE*) was performed using certified IPCE equipment (Enli Technology Co., Ltd. RC-BAS04). The *J-V* curves were measured under AM 1.5 G (100 mW cm^−2^) (Enli Technology Co., Ltd. SS-X50R). The *J-V* measurement signals were recorded by a Keithley 2400 source-measure unit.

## Results and Discussion

**Fig. 1:**
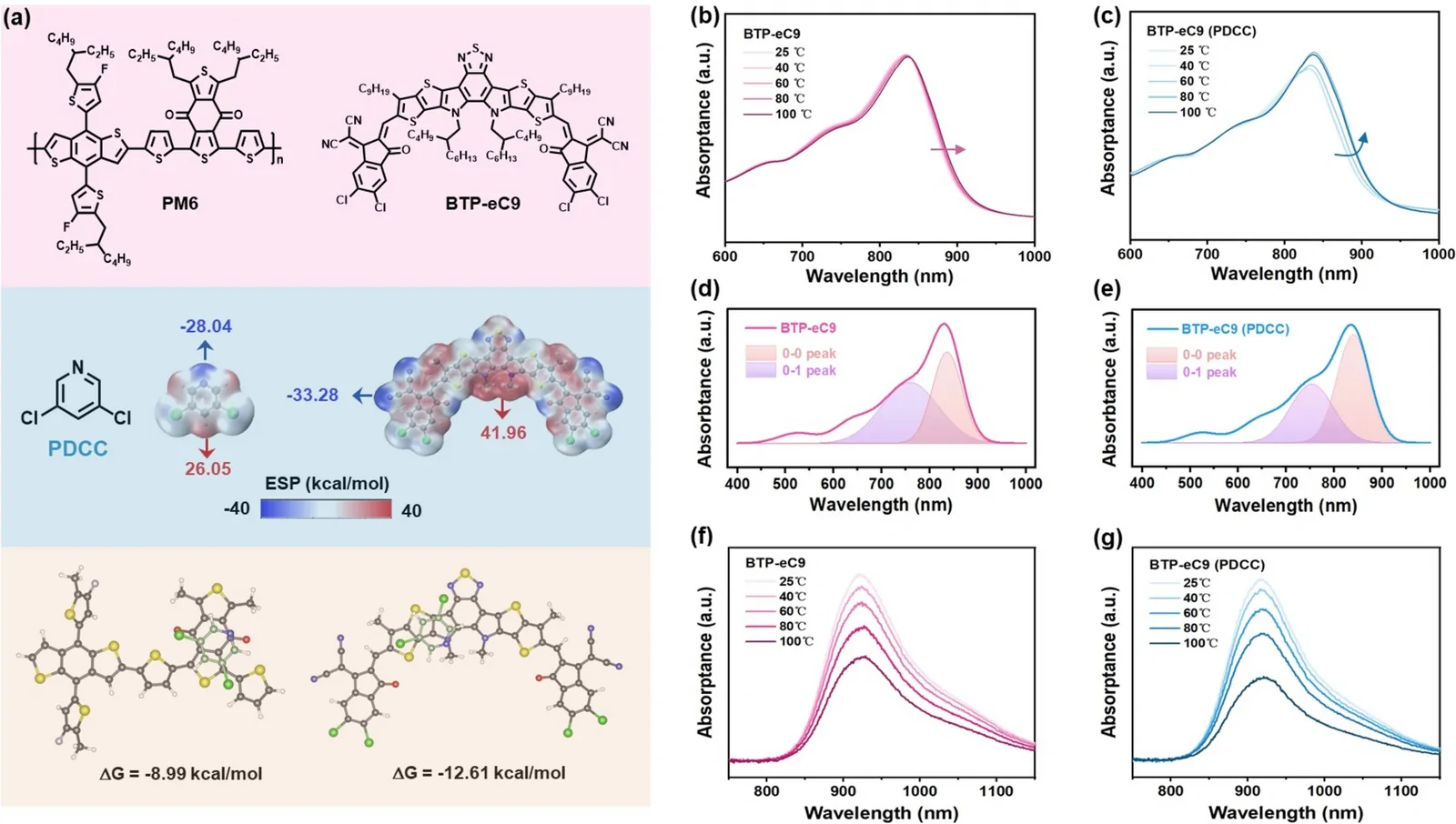
**a** Chemical structures of PM6, BTP-eC9, PDCC, ESP distribution and lowest-energy conformations calculated binding energies. Temperature-dependent UV–vis absorption of **b** BTP-eC9 and **c** PDCC-treated BTP-eC9 films. UV–vis absorption of **d** BTP-eC9 and **e** PDCC-treated BTP-eC9. Temperature-dependent PL of **f** BTP-eC9 and **g** PDCC-treated BTP-eC9 films

The molecular structures of the polymer donor (PM6), non-fullerene acceptor (BTP-eC9), solid additive (PDCC), along with the electrostatic potential (ESP) distribution of PDCC and binding energy (ΔE_b_) calculations, are illustrated in Figs. 1a and S1. As we can see, the PDCC exhibits a negative ESP distribution attributed to the high electronegativity of nitrogen, and BTP-eC9 with positive ESP distribution (BTP core units). Based on the theory of opposite polarity attraction, it is expected that there is a strong intermolecular interaction between PDCC and the BTP core units [32]. Furthermore, the ΔE_b_ calculations reveal that PDCC-treated BTP-eC9 exhibits a higher ΔE_b_ than PM6, indicating that PDCC preferentially interacts with the acceptor molecules [33], in line with the absorption results (Figs. S2 and S3). Generally, rational manipulation of intermolecular forces enables orderly aggregation behaviors, yielding enhanced crystallinity. To prove self-organization process of BTP-eC9 incorporated PDCC, temperature-dependent absorption was performed. As seen from Fig. 1b, d, both films show two distinct peaks: J-aggregation (0–0 peak)/monomer (0–1 peak) and progressive red-shift absorption with increasing temperature. In particular, the PDCC-processed film demonstrates a stronger red-shift along with increased peak intensity compared with the control, primarily attributed to the solid-to-gas phase transition of PDCC, which provides sufficient space for acceptor self-assembly, thereby enhancing J-aggregation of BTP-eC9. As evidenced by an increase in the A₀–₀/A₀–₁ ratio from 1.18 to 1.42 (Fig. 1d, e), a hallmark of efficiently self-assembly of acceptors and improved molecular ordering [34–37]. In addition, temperature-dependent photoluminescence (PL) spectra (Fig. 1f, g) further support this hypothesis. As expected, both films exhibit thermally responsive PL features, showing increasingly aggregated states under thermal annealing from 25 to 100 °C. Notably, the PDCC-treated BTP-eC9 results in more distinctly reduced intensity of PL spectrum, suggesting self-assembly and ordered aggregation happened. These results indicate that the incorporation of PDCC can enable acceptors with efficient self-assembly for ordered molecule stacking. Importantly, thermogravimetric analysis (TGA) and Fourier-transform infrared spectroscopy (FTIR) results (Figs. S4 and S5) confirm that the PDCC remain in films during spin-coating process, proving the self-assembly happen during thermal annealing. The synergistic effect of the PDCC–acceptor interactions and the transient spatial reorganization during PDCC volatilization promotes self-organization for optimizing film formation dynamics and molecular stacking, leading to well-structured and phase separation and active layer morphology.

**Fig. 2:**
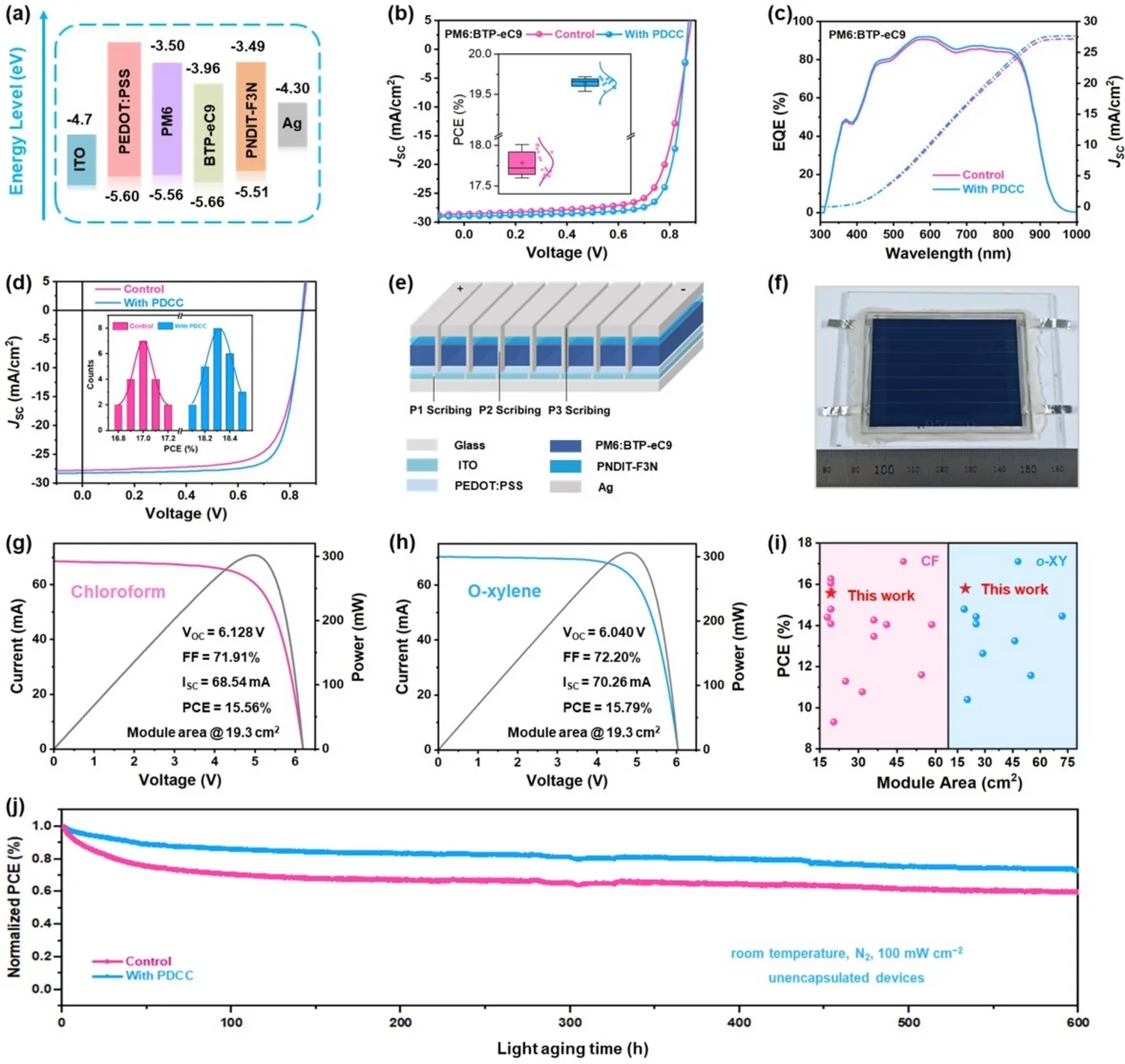
**a** Energy level of materials used in this work. **b** *J*-V, **c** EQE of control and PDCC-treated based PM6:BTP-eC9 OSCs processed with CF. **d** *J*-V curves of control and PDCC-treated devices processed with *o*-XY. **b** Device structure diagram and photograph of the large-area modules used in this work. **f** Photograph of the encapsulated large-area module. I-V and P–V curves of the large-area module processed with PDCC in **g** CF and **h** *o*-XY solvent. **i** The development of PCEs with CF and *o*-XY solvent for module area over 18 cm^2^ [38–57]. **j** Light stability of control and PDCC-induced devices

To investigate the photovoltaic performance of devices treated with PDCC, a standard architecture of ITO/PEDOT:PSS/active layer/PNDIT-F3N/Ag was utilized, with detailed experimental procedures and device optimization outlined in the Supporting Information (Table S1). The energy levels of materials used in this work present in Fig. 2a and are well-matched across each layer. Figure 2b depicts the current density–voltage (*J–V*) characteristics of OSCs based on PM6:BTP-eC9 without and with PDCC. The control device (PM6:BTP-eC9) achieved a PCE of 18.01%, with a voltage (*V*_OC_) of 867.6 mV, a current density (*J*_SC_) of 28.58 mA cm^−2^, and a FF of 72.64%. Remarkably, the PDCC-treated device reached a higher PCE of 19.72%, with significantly improved FF and *J*_SC_ of 78.81% and 28.96 mA cm^−2^ (Table 1). The same tendency exhibited in PM6:L8-BO (18.23% vs. 20.47%) and PM6:Y6 (16.98% vs. 18.13%) systems with remarkable improvements in FF (Tables 1 and S2, Figs. S6 and S8), reflecting excellent universality. The external quantum efficiency (EQE) spectra of the OSCs are illustrated in Fig. 2c. In the wavelength range of 450 to 850 nm, the EQE of the PDCC-treated device slightly surpassed that of the control device, leading to a higher *J*_SC_. The *J*_SC_ values derived from the EQE spectra were 27.80/28.17 mA cm^−2^ for the control/PDCC-treated PM6:BTP-eC9 OSCs, respectively, consistent with the *J*_SC_ values measured from the solar simulator (Table 1). The same tendency is also shown in other systems (Fig. S7 and Table S2). Device stability is as important as efficiency, to explore the impact of the PDCC on the device stability, the photo and thermal stability were systematically investigated. The light stability was recorded at 100 mW cm^−2^ in room temperature with N_2_ atmosphere. As shown in Fig. 2g, the PDCC-treated devices with better light stability, the PCE of control devices maintained 60.2% of the initial PCE were observed after 600 h, which is lower than that of devices processed with PDCC (maintaining 72.9% of the initial PCE). In addition, the PDCC-treated devices have better thermal stability (Fig. S9). These results highlight the significant potential of PDCC incorporation in enhancing efficiency and stability, attributed to effectively self-assembly of acceptors.

**Table 1:** Summary of photovoltaic performance of control and PDCC-treated OSCs under simulated AM 1.5G illumination (100 mW cm^−2^)

Condition	*V*_OC_ (mV)	FF (%)	*J*_SC_ (mA cm^-2^)	^*a)*^*J*_SC_^Cal^ (mA cm^−2^)	^*b)*^PCE (%)
PM6:BTP-eC9	867.6 (863.8 ± 3.7)	72.64 (71.58 ± 0.54)	28.58 (28.09 ± 0.35)	27.80	18.01 (17.85 ± 0.14)
PM6:BTP-eC9 (PDCC)	864.1 (861.5 ± 2.4)	78.81 (77.65 ± 0.92)	28.96 (28.61 ± 0.26)	28.17	19.72 (19.56 ± 0.16)
PM6:L8-BO	912.8 (911.3 ± 1.0)	73.25 (72.23 ± 0.84)	27.26 (27.01 ± 0.17)	26.29	18.23 (18.01 ± 0.14)
PM6:L8-BO (PDCC)	906.8 (905.3 ± 1.0)	82.16 (81.68 ± 0.38)	27.48 (27.37 ± 0.13)	26.55	20.47 (20.24 ± 0.11)

^*a)*^The *J*_SC_ calculated from the integrated EQE spectra. ^*b)*^ Statistical data obtained from at least 15 devices

In addition, PDCC-driven self-assembly strategy also has great potential in fabricating green-solvent-processed OSCs and large-area modules. Figure 2d shows the *J*-V curves and PCE distribution of control and PDCC-treated devices processed with *o*-XY solvent. The PDCC-treated device significantly enhances the PCE from 17.14% to 18.52%, with improved FF (72.55% vs. 77.49%) and *J*_SC_ (27.76 vs. 28.21 mA cm^−2^) (Table 2), the optimization process listed in Table S3. Similarly, the PDCC incorporated has excellent universality in other systems prepared with *o*-XY solvent (Table S4). Scaling up active layer fabrication presents a major morphological control challenge, due to CF with high volatility. Thus, we employed a higher-boiling and green solvent (*o*-XY) to fabricate large-scale OPV modules, which can shorten progress in industrial scalability. As shown in Fig. 2h, a well-optimized PDCC-treated module comprises seven sub-cells in series with an active area of 19.3 cm^2^ and a geometric fill factor (GFF) of 97.0%. The encapsulated large-area module is presented in Fig. 2i, optimization process is presented in Tables S5 and S6. Interestingly, a higher PCE of 15.79% was achieved, induced with PDCC in *o*-XY solvent, compared with control (13.87%) and CF as solvent (15.56%), shown in Figs. 2g, h and S14. Primarily incorporating PDCC can enhance the *J*-aggregation of acceptors, resulting in efficient self-assembly during the preparation of large-area modules, which is conducive to obtaining high-quality active layers. Particularly for high-boiling-point solvents, the regulation of molecular self-assembly proves more effective in large-scale fabrication processes. Figure 2f summarizes the PCE of OSCs modules based on CF and *o*-XY as solvents with an area over 18 cm^2^ [38–57]. It is worth noting that the PDCC-treated large-area modules have high efficiency, which confirm great potential of PDCC solid additive for fabricating large-area modules.

**Table 2:** Summary of photovoltaic performance of green-solvent-processed control and PDCC-treated PM6:BTP-eC9 OSCs under simulated AM 1.5G illumination (100 mW cm^−2^)

Condition	V_OC_ (mV)	FF (%)	*J*_SC_ (mA cm^−2^)	^*a)*^*J*_SC_^Cal^ (mA cm^−2^)	^*b)*^PCE (%)
Control (0.1 cm^2^)	851.0 (849.8 ± 1.1)	72.55 (71.67 ± 0.74)	27.76 (27.18 ± 0.69)	27.18	17.14 (17.00 ± 0.12)
PDCC (0.1 cm^2^)	846.9 (845.3 ± 1.2)	77.49 (76.77 ± 0.63)	28.21 (27.61 ± 0.59)	27.67	18.52 (18.31 ± 0.18)

Condition	V_OC_ (V)	FF (%)	*I*_SC_ (mA)	*J*_SC_^Cal^ (mA cm^−2^)	^*b)*^PCE (%)
Control (19.3 cm^2^)	6.12 (6.06 ± 0.05)	68.04 (66.17 ± 1.63)	64.53 (62.31 ± 2.12)	-	13.87 (13.58 ± 0.16)
PDCC (19.3 cm^2^)	6.04 (5.93 ± 0.08)	72.20 (70.34 ± 1.70)	70.26 (68.20 ± 2.07)	-	15.79 (15.56 ± 0.19)

^*a)*^ The *J*_SC_ calculated from the integrated EQE spectra. ^*b)*^ Statistical data obtained from at least 15 devices

**Fig. 3:**
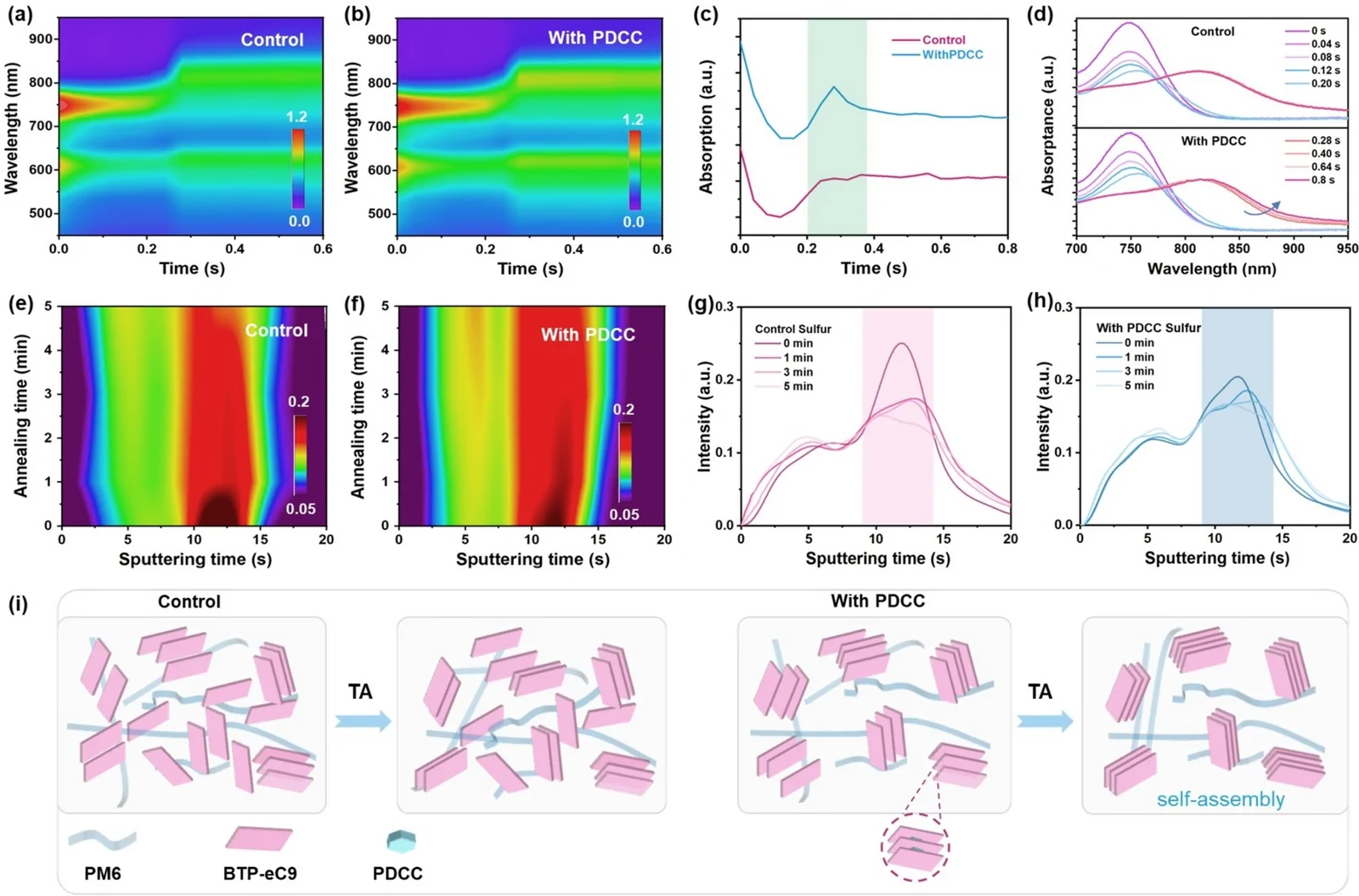
**a** and **b** In situ UV–Vis absorption line-cut color images. **c** Function of spin-coating time. **d** Line-cut profiles of the corresponding in situ UV–Vis absorption 2D data. **e** and **f** In situ GD-OES of sulfur element distribution upon thermal annealing color images. **g** and **h** Line-cut profiles of the corresponding in situ GD-OES 2D data of control and PDCC-treated blend films. **i** Schematic diagram of the film-formation mechanism of Y-series NFAs self-assembly induced by PDCC

To further gain insight into the effect of PDCC on the self-assembly behavior of Y-series NFAs molecules, the in situ absorption spectroscopy was employed to monitor the film formation process from solution to thin film state under PDCC treatment, corresponding 2D mapping images shown in Fig. 3a, b. By tracking the evolution of peak absorption wavelengths during spin-coating (Fig. 3c) can provide insights into molecular aggregation dynamics in blend processed with PDCC. As shown in Fig. 3c, the absorption of blend treated with PDCC initially increases and subsequently decreases (0.2–0.4 s), attributing to facilitated molecular self-assembly treated with PDCC, which is benefit to achieve ordered aggregation. In addition, a pronounced redshift was observed in both blends (Fig. 3d, e), which can be attributed to molecular stacking resulting from the phase transition from solution to solid state. Notably, the PDCC-treated blend demonstrated prolonged evolution toward absorption saturation (Fig. 3e), indicative of an extended and controlled self-assembly process. Furthermore, the in situ glow discharge optical emission spectroscopy (GD-OES) is employed to track the positional changes of Sulfur (S) upon thermal annealing [58]. Since both of donor and acceptor contain S element, the initial S distribution reflects the vertical arrangement of blend film. Moreover, the donor-rich bottom interface exhibits substantially higher S content than the acceptor-dominated top interface, this observation reflects the initial aggregation state of donor and acceptor molecules during the early stages of film formation. As shown in 2D mapping images (Fig. 3f, g), the amount of S element in the PDCC-treated blend is apparently less than that of control at the beginning annealing of 0–1 s. With the annealing time increasing, the distribution of S elements in PDCC-treated film changes slowly (Fig. 3i), while the control film changes abruptly (Fig. 3h), indicating slower film growth and improved donor/acceptor self-assembly. These results indicate that the PDCC-treated blend film can achieve ordered molecular stacking under thermal annealing, owing to the self-assembly behavior of molecules. These in situ results demonstrate that the incorporation of PDCC promotes molecular self-assembly, facilitating the formation of well-ordered molecular packing. The relevant film-forming mechanism diagram is shown in Fig. 3i.

**Fig. 4:**
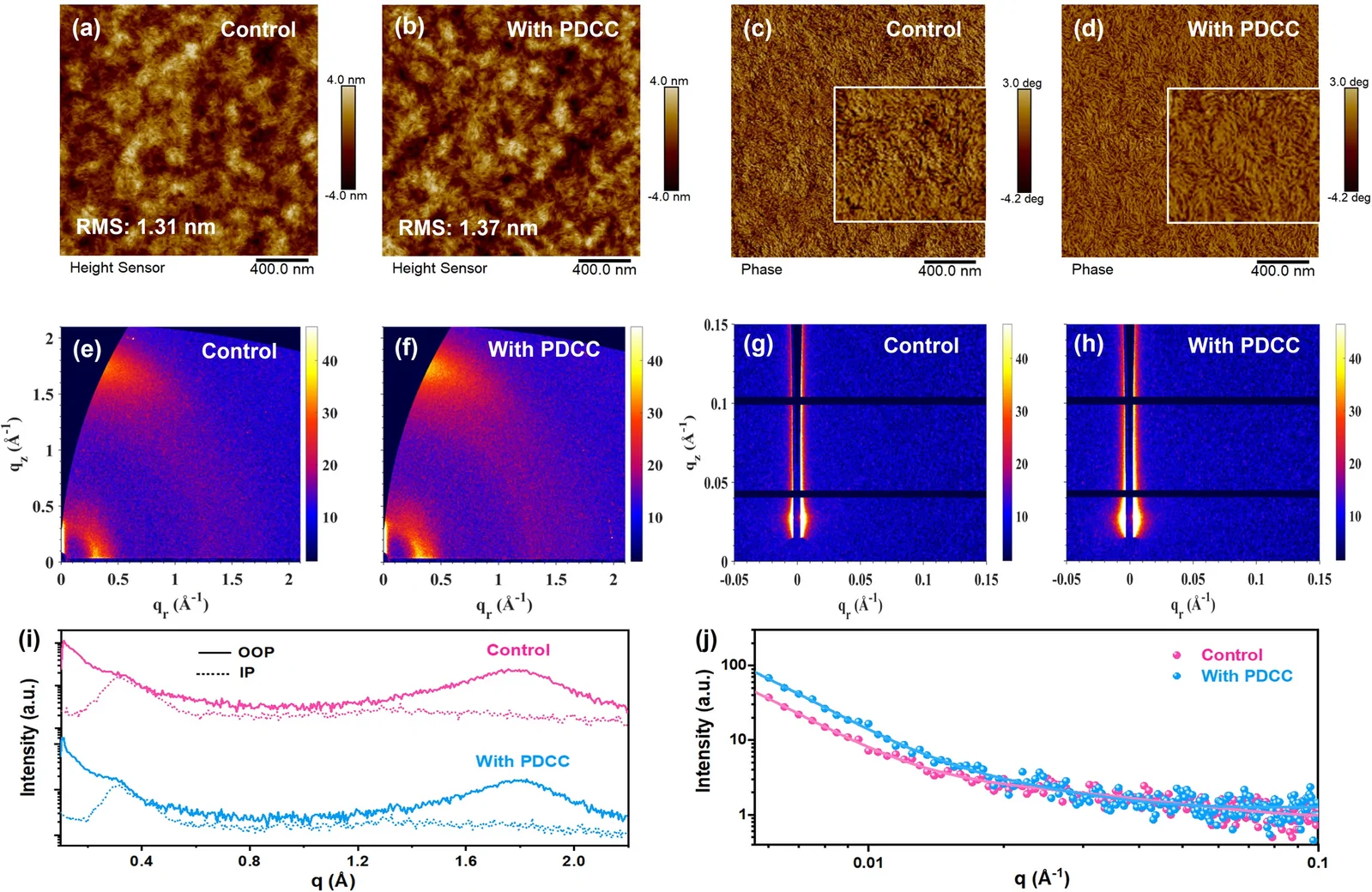
**a**–**d** AFM height and phase images of control and PDCC-treated samples. **e** and **f** 2D GIWAXS, **g** and **h** 2D GISAXS images of control and PDCC-treated blend films. **i** Line-cut profiles of the corresponding two-dimensional GIWAXS data, **j** GISAXS intensity profiles (symbols) and the best fitting (solid lines) along the in-plane direction of control and PDCC-treated blend films

The active layer morphology, donor/acceptor phase separation, and molecular aggregation optimized through PDCC-driven self-assembly strategy were examined using atomic force microscopy (AFM). As shown in AFM images (Fig. 4a–d), the PDCC-treated blend film exhibited a higher root mean square roughness (Rq) of 1.37 nm than the control (Rq = 1.31 nm), indicating that PDCC incorporation can finely adjust molecular aggregation. Furthermore, the PDCC-induced blend film displayed more distinct fiber and phase separation than the control film (Fig. 4c, d), facilitating efficient charge transport. The crystalline and molecular packing of the PDCC-treated films, assessed by Grazing-incidence wide-angle X-ray scattering (GIWAXS), are summarized in Fig. 4e, f and Table S7. Both blends showed similar molecular orientations, but the PDCC-treated films exhibited more pronounced diffraction peaks than the control films. The π-π stacking peak (010) at 1.773 Å^−1^ in the q_z_ direction for the PDCC-treated PM6:BTP-eC9 was more prominent than in the control blend, suggesting that PDCC promotes ordered molecular stacking. Additionally, the PDCC incorporation resulted in a larger coherence length (CCL) of 20.74 Å and smaller d-spacing (3.54 Å) compared to the control sample with a CCL of 18.83 Å and *d*-spacing of 3.55 Å, indicating improved molecular crystallinity and stacking. Enhanced crystallinity and more ordered stacking contribute to long-term device stability and efficiently charge transport in PDCC-treated blends.

Grazing incident small angle X-ray scattering (GISAXS) was used to assess D/A phase separation. The corresponding IP intensity plots and fitting results based on the Debye-Anderson-Brumberger (DAB) model and fractal-like network are shown in Fig. 4 g, h. X_DAB_ refers to the intermixing D/A domain size, and 2R_g_ represents the average pure domain size of the acceptor phase for the control and PDCC-treated PM6:BTP-eC9 samples. The calculated results for X_DAB_ and 2R_g_ are summarized in Table S5. The X_DAB_ and 2R_g_ of PDCC-treated blend films are 39 nm and 11 nm, larger than the control blend film (X_DAB_ ≈ 31 nm and 2R_g_ ≈ 9 nm), indicating improved D/A phase separation. These results are consistent with the AFM and GIWAXS study, which is benefit to charge transport for reaching a highly efficient device.

**Fig. 5:**
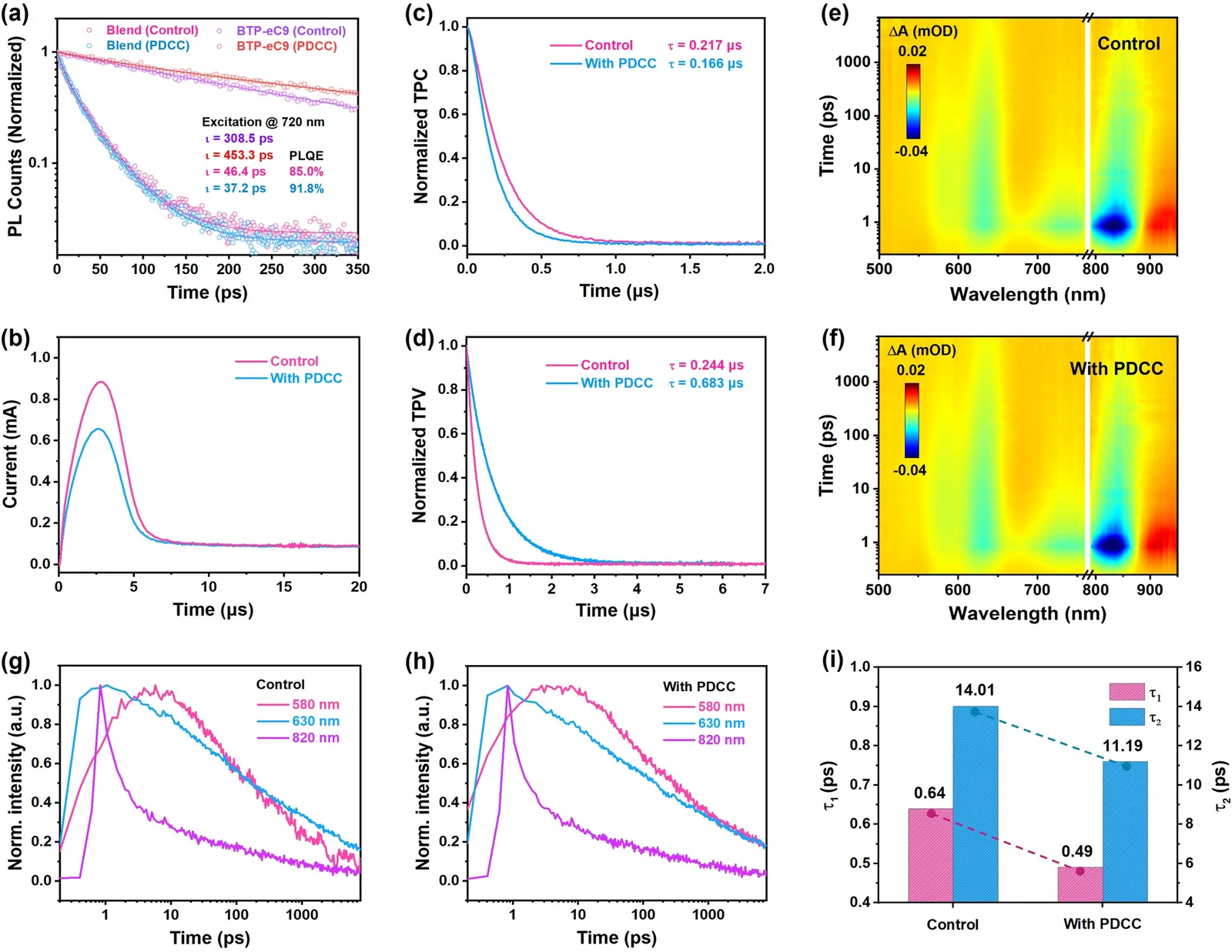
**a** TRPL kinetics of BTP-eC9 neat and blend films of control and PDCC-treated samples tracked at the peak position of the PL, and fits to the experimental data using a bi-exponential decay (solid line). **b** photo-CELIV curves, **c** TPC and **d** TPV, **e** and **f** 2D TA spectra, **g** and **h** decay dynamics probed at different wavelengths, **i** decay fitting time of control and PDCC-treated blends

The exciton dynamics of blend films without and with PDCC were measured using time-resolved photoluminescence (TRPL) under pulsed photoexcitation at 720 nm. The PL quenching efficiency (PLQE) was evaluated. The corresponding normalized TRPL of the neat and blend films are shown in Fig. 5a. The PDCC incorporation reduced the PL lifetime of blend films from 46.4 to 37.2 ps, resulting in enhanced PL quenching efficiency (PLQE) from 85.0% to 91.8%. Here, PLQE is calculated as (1-*τ*_blend_/*τ*_neat_), where τ_blend_ and τ_neat_ are the lifetimes of the blend and neat materials [31, 59]. These results align with the improved *J*_*SC*_, indicating PDCC's positive impact on exciton dissociation. Subsequently, the carrier mobilities were extracted from the photo-induced charge-carrier extraction in linearly increasing voltage (photo-CELIV) measurements (Fig. 5b). The control and PDCC-treated devices exhibited the carrier mobilities of 7.32 and 9.58 × 10^−4^ cm^2^ V^−1^ s^−1^, respectively. In addition, charge carrier transport properties were investigated using the space charge limited current (SCLC) method, with results shown in Figs. S10 and S11. The PDCC-treated samples demonstrated higher electron (6.06 × 10^−4^ cm^2^ V^−1^ s^−1^) and hole (2.05 × 10^−3^ cm^2^ V^−1^ s^−1^) mobilities compared to the control device with electron mobility of 4.41 × 10^−4^ cm^2^ V^−1^ s^−1^ and hole mobility of 1.68 × 10^−3^ cm^2^ V^−1^ s^−1^. Moreover, the hole/electron mobility ratio of the PDCC-induced sample is 3.38, closer to 1 compared to the control (3.81). Larger and more balanced mobilities in PDCC-treated devices result in higher FF.

Transient photovoltage/photovoltaic (TPV/TPC) measurements were employed to analysis charge recombination dynamics and carrier extraction across active layers. As seen from the TPC (Fig. 5c), the charge extraction times for control and PDCC-treated devices are 0.217 and 0.166 μs, respectively, indicating more efficient charge extraction in PDCC-treated devices. Carrier lifetimes (τ) under open-circuit conditions (Fig. 5d) were extracted from TPV decay dynamics using simple mono-exponential fits. The device with PDCC exhibits a longer τ value of 0.683 μs compared to the control device (τ = 0.244 μs), resulting in less recombination in PDCC-treated devices. Femtosecond transient absorption (fs-TA) spectroscopy further probed charge transfer in control and PDCC-treated blend films, and the TA spectra were measured with pump at 780 nm. The 2D spectrum and TAS profiles at indicated delay times are shown in Fig. S13. As we can see, the ground-state bleach (GSB) signals of BTP-eC9 appear at ~ 820 nm, while the excited-state absorption (ESA) features are observed near 920 nm in the decay traces. It is noting that with the decay of BTP-eC9 bleach peak (820 nm), the PM6 GSB peak at around 580 nm rises, suggesting the hole-transfer process from BTP-eC9 to PM6 (Fig. 5e–h). The fast/slow components (τ_1_ and τ_2_) were extracted by fitting a double exponential function to the kinetic signals around 580 nm. The resulting τ_1_/τ_2_ values for control and PDCC-treated blends were fitted to be 0.64/14.01 and 0.49/11.19 ps, shown in Fig. 5i. The reduced τ_1_ with the incorporation of PDCC indicates enhanced donor–acceptor interactions, thus facilitating efficient exciton dissociation and consequently enabling a higher *J*_SC_ in the PDCC-treated devices [60–63]. The shorter τ_2_ upon enhanced crystallization indicates higher diffusion constants, linked to better molecular stacking in line with GIWAXS results. These findings demonstrate that the incorporation of PDCC facilitates both exciton dissociation and diffusion processes, thereby accounting for enhanced *J*_SC_ and FF.

## Conclusions

In summary, we have successfully demonstrated a PDCC-driven self-assembly strategy that effectively regulates the self-assembly of Y-series NFAs during solvent evaporation and film formation process. This approach significantly enhances the performance and stability of OSCs, increasing the PCE of PM6:BTP-eC9 devices from 18.01% to 19.72% and PM6:L8-BO devices from 18.23% to 20.47%, while simultaneously improving light and thermal stability. Notably, when translated to sustainable fabrication, this strategy significantly boosts the PCE of large-area green-solvent-processed OSC modules (19.3 cm^2^) from 13.87% to 15.79%, ranking it among the best-performing green-solvent-processed large-area OSC modules (> 18 cm^2^). PDCC serves as a multifunctional additive in optimizing both efficiency and stability through three key mechanisms: (i) stronger intermolecular interactions between PDCC and NFAs, promoting J-aggregation of the acceptor phase during film formation, thereby enhancing *J*_SC_; (ii) more ordered molecule packing and improved phase-separation, leading to enhanced charge transport, suppressed recombination and improved FF; (iii) enhanced crystallinity and a more uniform self-assembly process, resulting in a stable phase-separation morphology, crucial for long-term device stability. This work establishes a promising strategy for advancing the industrial production of high-efficiency and stable OSCs, paving the way for their commercialization on a larger scale.

## Supplementary Information

Below is the link to the electronic supplementary material.

Supplementary file1 (DOCX 768 KB)
