# Supplementary material for: Self-Assembly Control of Y-Series Non-fullerene Acceptors for Sustainable and Scalable Organic Photovoltaics
Source: Nanomicro Lett. 2026 Jan 5;18:182. doi: 10.1007/s40820-025-02021-7 (PMC12765764; doi:10.1007/s40820-025-02021-7)
Supplement: Supplementary file 1 — Supplementary file1 (DOCX 768 KB) [file 40820_2025_2021_MOESM1_ESM.docx]

Supporting Information for

**Self-Assembly Control of Y-Series Non-Fullerene Acceptors for Sustainable and Scalable Organic Photovoltaics**

Dingqin Hu^1,2^, Hua Tang^3,4,5,*^, Jiehao Fu^6^, Yaohui Li^7^, Lei Liu^8^, Peihao Huang^8^, Jie Lv^6^, Daming Zheng^6^, Yakun He^5^, Heng Liu^9^, Baomin Xu^10^, Zheng Hu^11^, Xinhui Lu^9^, Zeyun Xiao^8^, Gang Li^6^, Yang (Michael) Yang^7^, Frédéric Laquai^5^, Christoph J. Brabec^3,4^, Duu-Jong Lee^2, *^, Hsien-Yi Hsu^1, *^

^1^ School of Energy and Environment, Department of Materials Science and Engineering, Centre for Functional Photonics (CFP), City University of Hong Kong, Kowloon Tong, Hong Kong, P. R. China

^2^ Department of Mechanical Engineering, City University of Hong Kong, Kowloon Tong, Hong Kong, P. R. China

^3^ Institute of Materials for Electronics and Energy Technology (i-MEET), Friedrich-Alexander-Universität Erlangen-Nürnberg, Martensstrasse 7, 91058 Erlangen, Germany

^4^ Helmholtz-Institute Erlangen-Nürnberg (HI ERN), Immerwahrstraße 2, 91058 Erlangen, Germany

^5^ KAUST Solar Center, Physical Sciences and Engineering Division (PSE), King Abdullah University of Science and Technology (KAUST), Thuwal, 23955-6900, Kingdom of Saudi Arabia

^6^ Department of Electronic and Information Engineering Research Institute for Smart Energy (RISE), The Hong Kong Polytechnic University Hung Hum, Kowloon, 999077, Hong Kong, P. R. China

^7^ State Key Laboratory of Modern Optical Instrumentation College of Optical Science and Engineering, Zhejiang University, Hangzhou, Zhejiang, 310027, P. R. China

^8^ Chongqing Institute of Green and Intelligent Technology, Chinese Academy of Sciences, Chongqing, 400714, P. R. China

^9^ Department of Physics, The Chinese University of Hong Kong, New Territories, Hong Kong 999077, P. R. China

^10^ Department of Materials Science and Engineering, Southern University of Science and Technology, Shenzhen 518055, P. R. China

^11^ Key Laboratory of Mesoscopic Chemistry of MOE and Jiangsu Provincial Laboratory for Nanotechnology, School of Chemistry and Chemical Engineering, Nanjing University, Nanjing 210023, P. R. China

*Corresponding authors. E-mail: [sam.hyhsu@cityu.edu.hk](mailto:sam.hyhsu@cityu.edu.hk) (Hsien-Yi Hsu); [tuclee@cityu.edu.hk](mailto:tuclee@cityu.edu.hk) (Duu-Jong Lee); [hua.tang@fau.de](mailto:hua.tang@fau.de) (Hua Tang)

**S1 Experimental Section**

**S1.1 Density Functional Theory Computation**

The molecular geometry optimizations of PM6, BTP-eC9 and PDCC are performed by Gaussian 1613 at B3LYP/6-31G* level, where the long alkyl side chains are simplified to methyl groups to save computational cost. Conformational searches of the complex (PM6 and PDCC, BTP-eC9 and PDCC) are performed to find the low-energy conformers. 300 conformers are generated and optimized by semi-empirical extended tight-binding method GFN2-xTB as implemented in xtb14 program. The conformers with the lowest energy are further optimized by Gaussian 16, where the 6-311G** basis set in conjunction with the D3BJ dispersion correction is used to calculate Gibbs free energy. The binding energy is determined by $\text{∆}\text{G}_{\text{b}}\text{=}\text{G}_{\text{com}}\text{-}\left( \text{G}_{\text{M}\text{1}}\text{+}\text{G}_{\text{M}\text{2}} \right)$, where$\text{G}_{\text{M}\text{1}}$, $\text{G}_{\text{M}\text{2}}$ and $\text{G}_{\text{com}}$ are the Gibbs free energy of molecule 1, molecule 2 and their complex.

**S1.2 The thermogravimetric analysis (TGA)**

The thermogravimetric analysis (TGA) was carried out on a Mettler Toledo TGA/DSC 1 thermogravimetric analyzer with a thermal balance under the protection of nitrogen.

**S1.3 Fourier-transform infrared spectroscopy (FTIR)**

The fourier-transform infrared spectroscopy (FTIR) of with and without PDCC samples were carried out on Perkin Elemer Spectrum 2.

**S1.4 Temperature-dependent Absorption**

The temperature-dependent UV-vis absorption spectra of BTP-eC9 and BTP-eC9 (PDCC) films were obtained via using a Shimadzu UV-3600 UV-VIS NIR spectrophotometer. The temperature gradients considered are 25, 40, 60, 80 and 100 ℃.

**S1.5 Temperature-dependent photoluminescence (PL)**

The temperature-dependent PL spectra of BTP-eC9 and BTP-eC9 (PDCC) films were obtained via using Edinburgh FLS980. The temperature gradients considered are 25, 40, 60, 80 and 100 ℃.

**S1.6 Atomic force microscopy (AFM)**

Topographic images of the films were obtained from a Bruker atomic force microscopy (**AFM**) with the type of dimension edge with Scan AsystTM in the tapping mode using an etched silicon cantilever at a nominal load of ~40 nN, and the scanning rate for a 2 μm×2 μm image size was 1.5 Hz.

**S1.7 GIWAXS/GISAXS**

GIWAXS measurements were carried out with a Xeuss 2.0 SAXS laboratory beamline using a Cu X-ray source (8.05 keV, 1.54 Å) and a Pilatus3R 300K detector. The incidence angle is 0.2°.

GISAXS measurements were carried out with a Xeuss 2.0 SAXS laboratory beamline using a Cu X-ray source (8.05 keV, 1.54 Å) and a Pilatus3R 300K detector. The incidence angle is 0.2°.

**S1.8** **Space-charge-limited current (SCLC)**

The carrier mobility (hole and electron mobility) of photoactive layer was determined by fitting the dark current of hole/electron-only diodes to the space-charge-limited current (SCLC) model. Hole-only diode configuration: Glass/ITO/PEDOT:PSS/active layer/MoO_3_/Ag; Electron-only diode configuration: Glass/ITO/ZnO/PNDIT-F3N/active layer/PNDIT-F3N/Ag. V_bi_=0 was used for both fittings. The active layer thickness was determined by a Tencor surface profilometer. The electric-field dependent SCLC mobility was estimated using the following equation:

$$\text{J}\left( \text{V} \right)\text{=}\frac{\text{9}}{\text{8}}\text{e}_{\text{0}}\text{e}_{\text{r}}\text{m}_{\text{0}}\text{exp}\left( \text{0.89b}\sqrt{\frac{\text{V}\text{-}\text{V}_{\text{bi}}}{\text{L}}} \right)\frac{\left( \text{V}\text{-}\text{V}_{\text{bi}} \right)^{\text{2}}}{\text{L}^{\text{3}}}$$

**S1.9 Time-resolved photoluminescence (TRPL)**

The TRPL samples were sealed in a vacuum chamber and excited at 720 nm with the fluence of 0.5 nJ/cm^2^. The wavelength-tunable Chameleon Ultra Laser (Coherent) was used together with a Compact OPO-VIS, and .the repetition rate of the fs pulses was 80 MHz. The PL of the samples was collected by an optical telescope (consisting of two plano-convex lenses) and focused on the slit of a spectrograph (PI Spectra Pro SP2300) and detected with a Streak Camera (Hamamatsu C10910) system with a temporal resolution of 1.4 ps. The data was acquired in photon counting mode using the Streak Camera software (HPDTA) and exported to Origin Pro 2020 for further analysis.

**S1.10 Time-resolved Transient Absorption Spectroscopy (fs-TAS)**

Femtosecond transient absorption (fs-TA) spectroscopy was employed to track the absorption changes in excited states over time, thereby elucidating the carrier dynamics on the femtosecond to nanosecond scale. The excitation source was an amplified Yb laser (1046 nm) that produced 283.9 fs pulses at a 2 kHz repetition rate. The laser power is 500 μJ, and the spectral range used is 480~950 nm. The output was divided into two beams; the more intense one served as the 1046 nm pump light, while the other was directed into a sapphire plate to create a broadband supercontinuum probe light. An optical chopper was used to adjust the pump pulses' repetition rate to 500 Hz, which were then focused on the sample in conjunction with the probe pulse (white light). The TA spectra were derived by comparing the probe light spectra with and without the pump light excitation. The photoinduced changes in absorption as a function of wavelength were represented in terms of optical density (OD) changes (ΔOD(λ)). By fine-tuning the delay between the pump and probe pulses, a 3D transient spectral image ΔOD (λ,t) was constructed.

**Supplementary Figures**


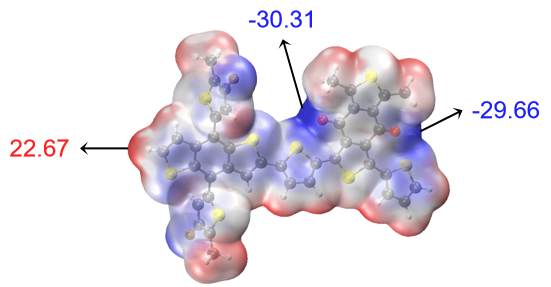


**Fig. S1** The ESP distribution of PM6


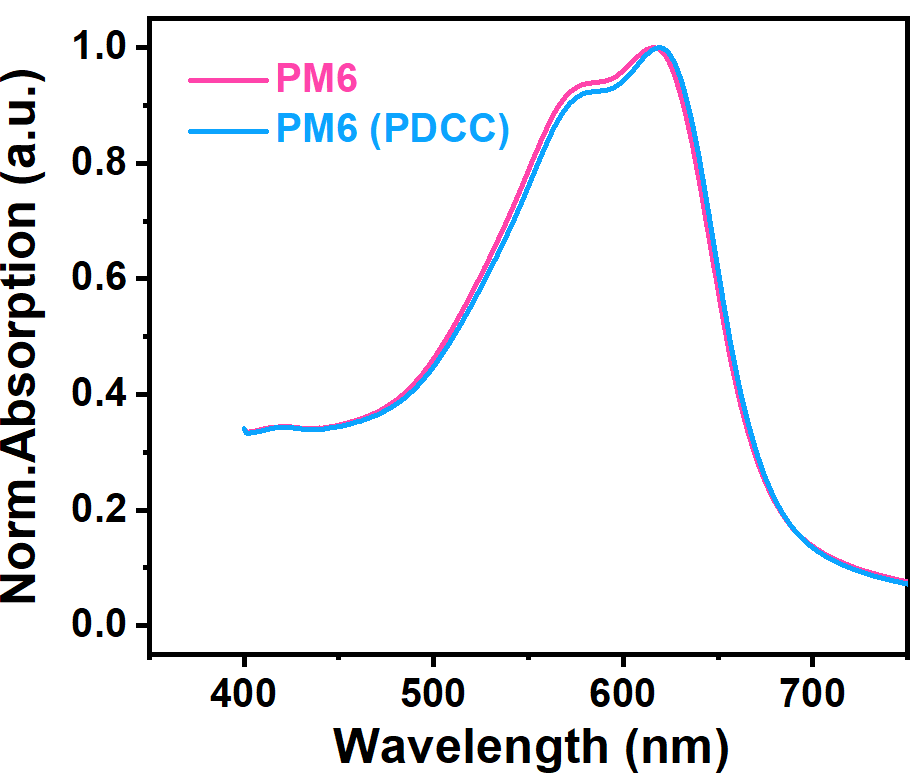


**Fig. S2** The absorption of PM6 and PM6 (PDCC)


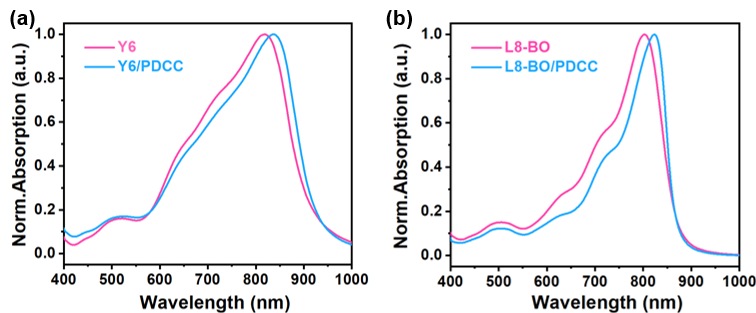


**Fig. S3** The absorption of (**a**) Y6 and PDCC treated Y6. (**b**) L8-BO and PDCC treated L8-BO


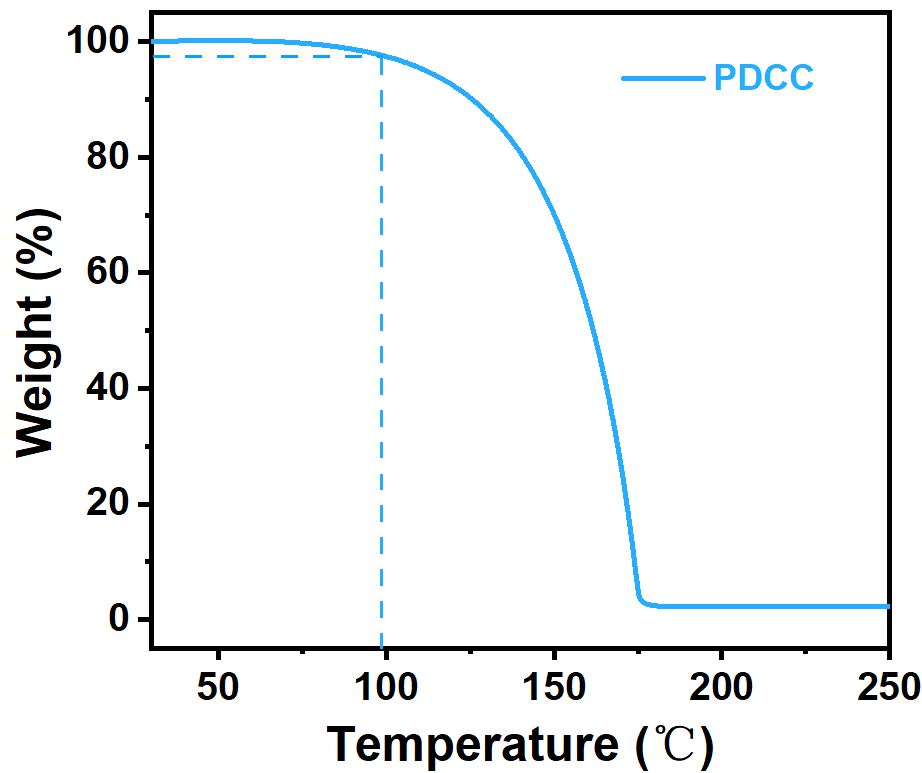


**Fig. S4** TGA plot of PDCC at a scan rate of 10 °C min^−1^


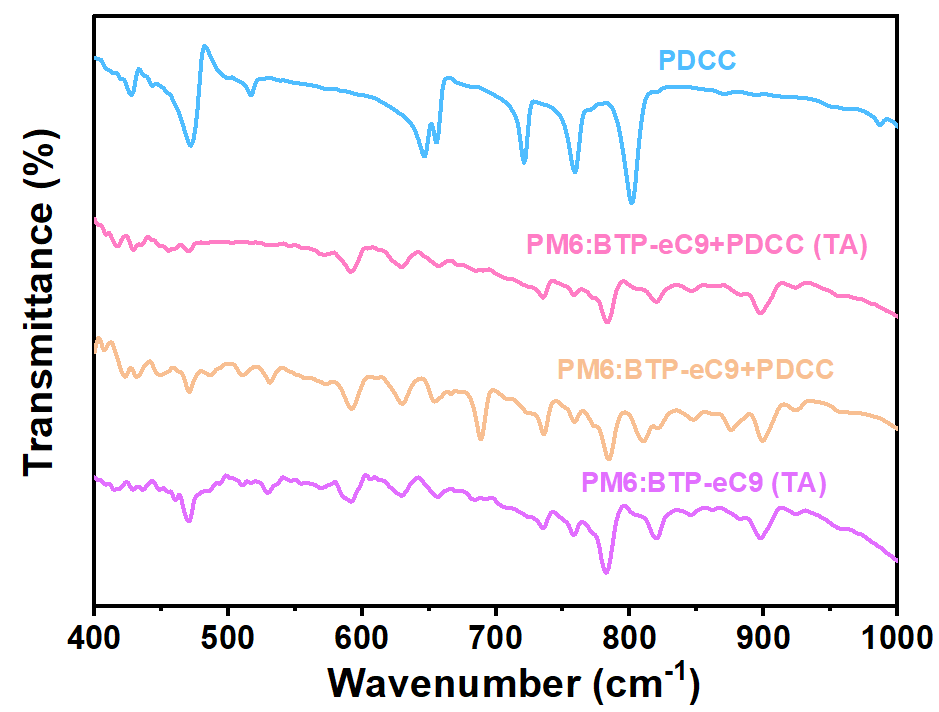


**Fig. S5** FT-IR spectra of the PDCC, PM6:BTP-eC9 and PM6:BTP-eC9 (PDCC) blends with thermal annealing


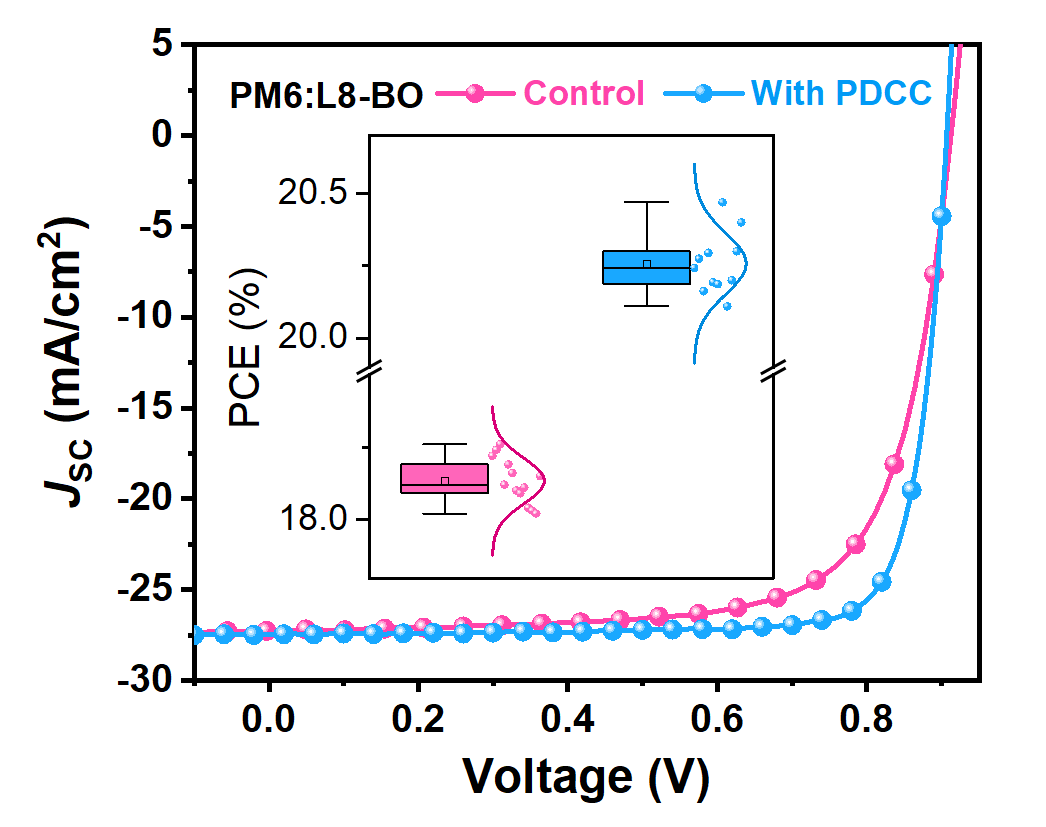


**Fig. S6** The *J*-V curves of PM6:L8-BO in PDCC induced devices with CF solvent


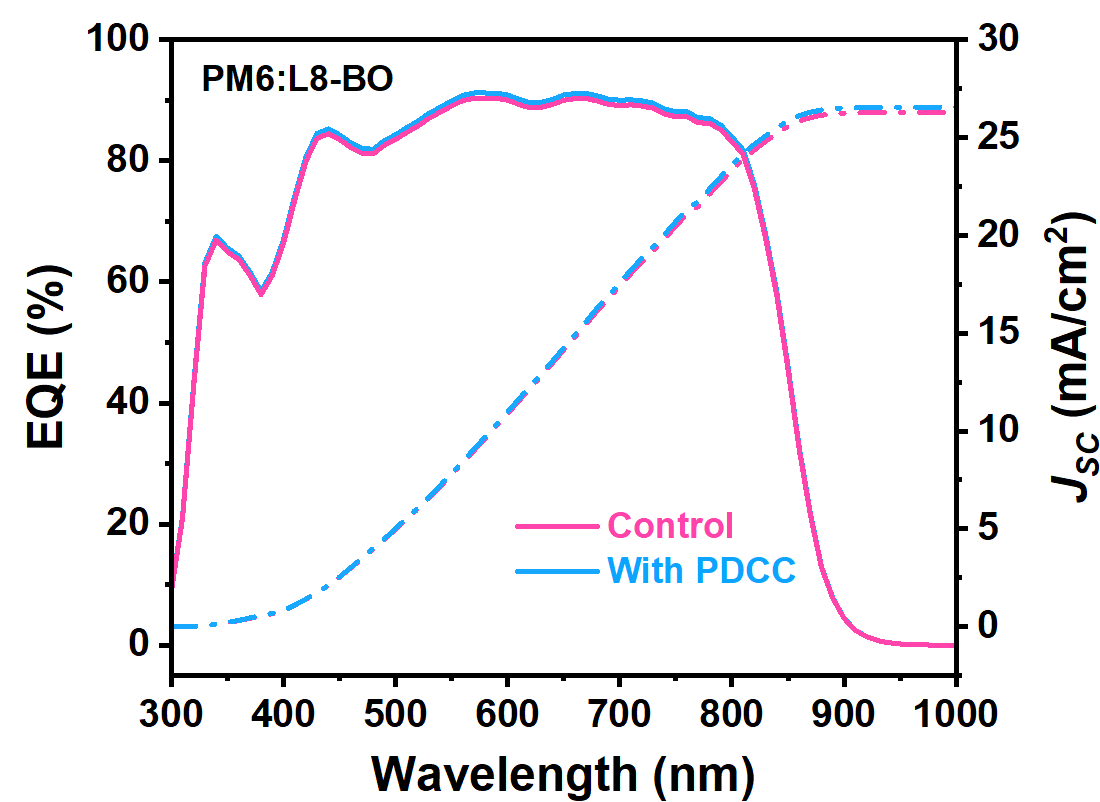


**Fig. S7** The EQE of PM6:L8-BO in PDCC induced devices with CF solvent


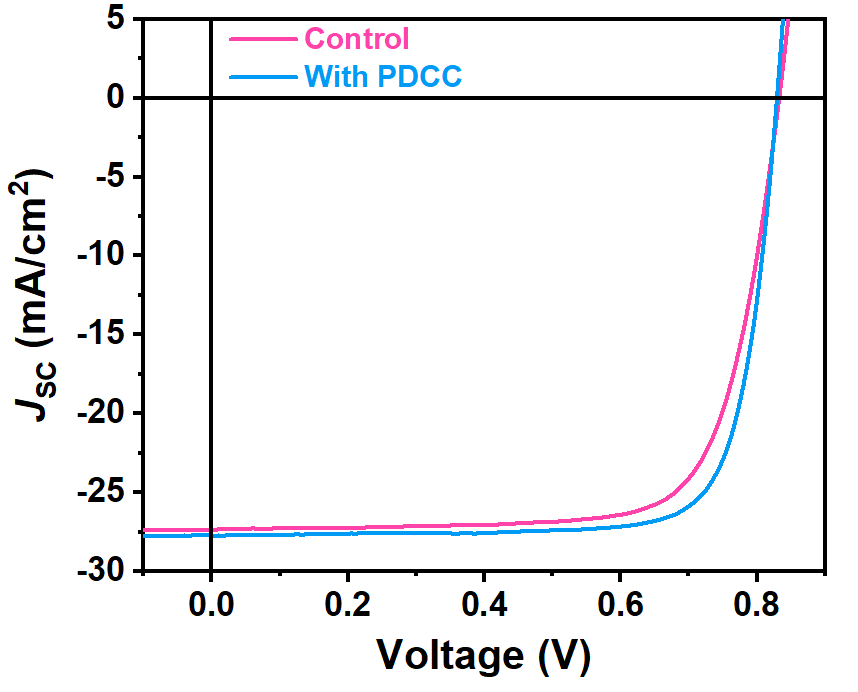


**Fig. S8** The *J*-V curves of PM6:Y6 in PDCC induced devices with CF solvent


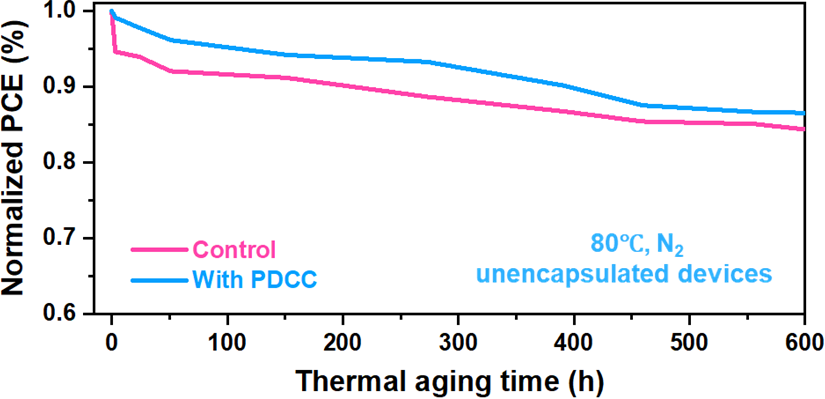


**Fig. S9** The thermal stability of control and PDCC induced unencapsulated devices under 80℃ treatment in glovebox


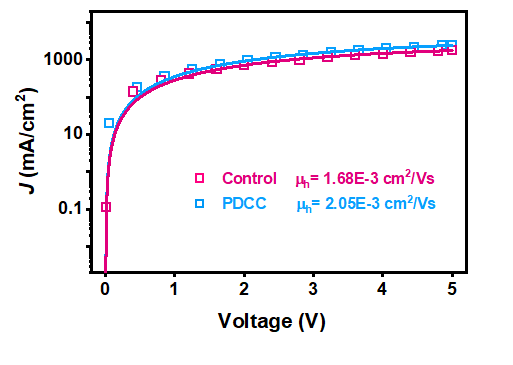


**Fig. S10** The hole mobility of PM6:BTP-eC9 w/o and with PDCC


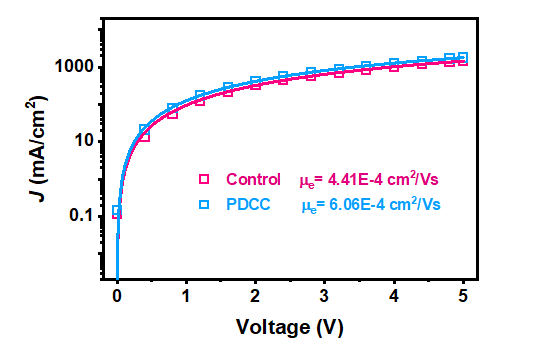


**Fig. S11** The electron mobility of PM6:BTP-eC9 w/o and with PDCC


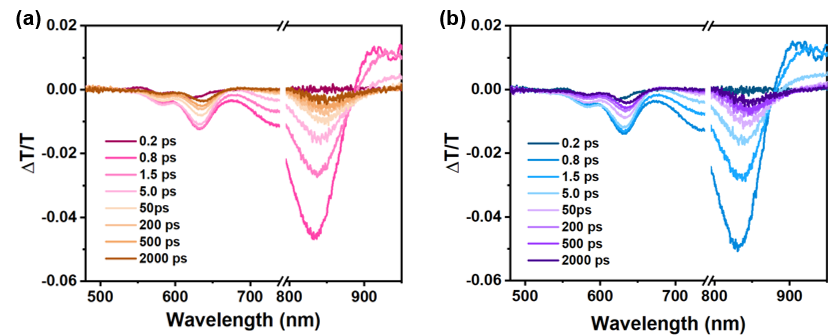


**Fig. S12** Line-cuts of fs-TAS at indicated delay times


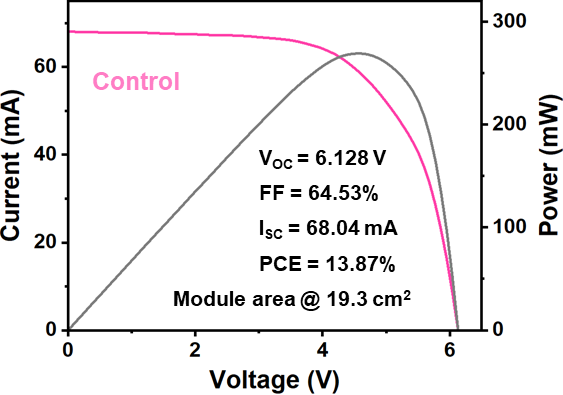


**Fig. S13** I-V and P-V curves of the large-area module based PM6:BTP-eC9 in *o*-XY solvent

**Supplementary Tables**

**Table S1** The photovoltaic performance in PM6:BTP-eC9 (CF) systems with different adding of PDCC.

| **Concentration** | **V_OC_ (mV)** | **FF (%)** | ***J*_SC_ (mA/cm^2^)** | **PCE (%)** |
| --- | --- | --- | --- | --- |
| 0 mg/mL | 867.6 | 72.64 | 28.58 | 18.01/17.85 |
| 4 mg/mL | 866.6 | 76.94 | 28.87 | 19.25/19.10 |
| 6 mg/mL | 865.4 | 78.43 | 28.88 | 19.60/19.44 |
| 8 mg/mL | 864.1 | 78.81 | 28.96 | 19.72/19.56 |
| 10 mg/mL | 863.2 | 77.92 | 28.62 | 19.25/19.04 |

**Table S2** The photovoltaic performance in PDCC induced devices with CF solvent

| **Active layer** | **V_OC_ (mV)** | **FF (%)** | ***J*_SC_ (mA/cm^2^)** | **PCE (%)** |
| --- | --- | --- | --- | --- |
| PM6:Y6 | 832.3 | 74.59 | 27.35 | 16.98 |
| PM6:Y6 (PDCC) | 829.2 | 78.82 | 27.73 | 18.13 |

**Table S3** The photovoltaic performance in PM6:BTP-eC9 (*o*-XY) systems with different adding of PDCC

| **Concentration** | **V_OC_ (mV)** | **FF (%)** | ***J*_SC_ (mA/cm^2^)** | **PCE (%)** |
| --- | --- | --- | --- | --- |
| 0 mg/mL | 851.0 | 72.55 | 27.76 | 17.14/17.00 |
| 4 mg/mL | 850.6 | 73.41 | 27.78 | 17.35/17.19 |
| 6 mg/mL | 848.7 | 75.39 | 27.96 | 17.89/17.73 |
| 8 mg/mL | 846.9 | 77.49 | 28.21 | 18.52/18.31 |
| 10 mg/mL | 845.3 | 77.12 | 28.10 | 18.32/18.11 |

**Table S4** The photovoltaic performance in PDCC induced devices with *o*-XY solvent

| **Active layer** | **V_OC_ (mV)** | **FF (%)** | ***J*_SC_ (mA/cm^2^)** | **PCE (%)** |
| --- | --- | --- | --- | --- |
| PM6:Y6 | 817.7 | 73.26 | 26.65 | 15.96 |
| PM6:Y6 (PDCC) | 814.6 | 76.97 | 27.01 | 16.94 |
| PM6:L8-BO | 871.9 | 72.65 | 26.02 | 16.48 |
| PM6:L8-BO (PDCC) | 862.3 | 76.57 | 26.60 | 17.56 |

**Table S5** Photovoltaic performance of green-solvent-processed PDCC-treated PM6:BTP-eC9 OSCs with different spin-coating speed under simulated AM 1.5G illumination (100 mW cm^-2^)

| **Active layer** | **Condition** | **V_OC_ (V)** | **FF (%)** | ***I*_SC_ (mA)** | **PCE (%)** |
| --- | --- | --- | --- | --- | --- |
| With PDCC  19.3 cm^2^ | 2500 r/s | 6.13 | 70.18 | 64.41 | 14.27 |
|  | 3000 r/s | 6.13 | 70.49 | 67.95 | 15.13 |
|  | 3500 r/s | 6.13 | 71.55 | 68.50 | 15.48 |
|  | 4000 r/s | 6.13 | 71.50 | 68.38 | 15.44 |

**Table S6** Photovoltaic performance of green-solvent-processed PDCC-treated PM6:BTP-eC9 OSCs with and without thermal annealing under simulated AM 1.5G illumination (100 mW cm^-2^)

| **Active layer** | **Condition** | **V_OC_ (V)** | **FF (%)** | ***I*_SC_ (mA)** | **PCE (%)** |
| --- | --- | --- | --- | --- | --- |
| With PDCC  19.3 cm^2^ | w/o TA | 6.13 | 70.17 | 64.35 | 14.26 |
|  | TA, 90 ℃ | 6.04 | 71.95 | 70.39 | 15.76 |

**Table S7** Analysis data of the two-dimensional GIWAXS/GISAXS results in out-of-plane direction (OOP) of control and PDCC induced blend films

| Blend films | Lattice plane | Peak location (A^-1^) | d-spacing (Å) | Coherence length (Å) | 2Rg (nm) | X_AB_ (nm) |
| --- | --- | --- | --- | --- | --- | --- |
|  |  | q_z_ | q_z_ | q_z_ |  |  |
| Control | 010 | 1.768 | 3.55 | 18.93 | 9 | 31 |
| PDCC | 010 | 1.773 | 3.54 | 20.74 | 11 | 39 |
